# A questionnaire-based survey on 3D image-guided brachytherapy for cervical cancer in Japan: advances and obstacles

**DOI:** 10.1093/jrr/rrv047

**Published:** 2015-08-11

**Authors:** Tatsuya Ohno, Takafumi Toita, Kayoko Tsujino, Nobue Uchida, Kazuo Hatano, Tetsuo Nishimura, Satoshi Ishikura

**Affiliations:** 1 Gunma University Heavy Ion Medical Center, Gunma University, 3-39-22 Showa-machi, Maebashi, Gunma, 371–8511, Japan; 2 Department of Radiology, Graduate School of Medical Science, University of the Ryukyus, 207 Uehara, Nishihara-cho, Okinawa, 903–0215, Japan; 3 Department of Radiation Oncology, Hyogo Cancer Center, 13–70 Kitaoji-cho, Akashi, Hyogo, 673–8558, Japan; 4 Department of Radiation Oncology, Tottori University Hospital, Nishi-cho, Yonago, Tottori, 683–8504, Japan; 5 Tokyo Bay Advanced Imaging and Radiation Oncology Clinic MAKUHARI, 1–17 Toyosuna, Mihama-ku, Chiba, 261–0024, Japan; 6 Division of Radiation Oncology, Shizuoka Cancer Center, 1007 Shimonagakubo, Nagaizumi-cho, Shizuoka, 411–8777, Japan; 7 Department of Radiology, Koshigaya Municipal Hospital, 10-47-1 Higashikoshigaya, Koshigaya, Saitama 343–8577, Japan

**Keywords:** image-guided brachytherapy, cervical cancer, 3D planning, questionnaire-based survey, high-dose-rate brachytherapy

## Abstract

The purpose of this study is to survey the current patterns of practice, and barriers to implementation, of 3D image-guided brachytherapy (3D-IGBT) for cervical cancer in Japan. A 30-item questionnaire was sent to 171 Japanese facilities where high-dose-rate brachytherapy devices were available in 2012. In total, 135 responses were returned for analysis. Fifty-one facilities had acquired some sort of 3D imaging modality with applicator insertion, and computed tomography (CT) and magnetic resonance imaging (MRI) were used in 51 and 3 of the facilities, respectively. For actual treatment planning, X-ray films, CT and MRI were used in 113, 20 and 2 facilities, respectively. Among 43 facilities where X-ray films and CT or MRI were acquired with an applicator, 29 still used X-ray films for actual treatment planning, mainly because of limited time and/or staffing. In a follow-up survey 2.5 years later, respondents included 38 facilities that originally used X-ray films alone but had indicated plans to adopt 3D-IGBT. Of these, 21 had indeed adopted CT imaging with applicator insertion. In conclusion, 3D-IGBT (mainly CT) was implemented in 22 facilities (16%) and will be installed in 72 (53%) facilities in the future. Limited time and staffing were major impediments.

## INTRODUCTION

Recent patterns of care for brachytherapy have demonstrated that gynecological brachytherapy remains the most common application of this technology, although with regional and community-specific variations [1, 2]. Since publication of the concept and terms of 3D image-guided brachytherapy (3D-IGBT) for cervical cancer in 2005, a common approach for ensuring target coverage and avoiding excessive exposure to organs at risk has been developed [3, 4]. Regarding dose–volume histogram (DVH) parameters in particular, a dose–response relationship has been reported between cervical tumor control and D90 for the high-risk clinical target volume (HR-CTV), as well as between late complications and D2cm^3^ for the rectum and bladder [5, 6]. Recent clinical data using MRI-guided adaptive brachytherapy have shown excellent 3-year local control rates of 95–100% for limited/favorable disease (Stages IB/IIB) and 85–90% for large/poor response disease (Stages IIB/III/IV), with acceptable treatment-related morbidities [7].

Advances in image guidance for applicator insertion and treatment planning seem to have influenced clinical practice in brachytherapy for cervical cancer. However, reports regarding practice patterns have been scarce worldwide. Although the results of several surveys on the practice of 3D-IGBT have been published by various study groups worldwide [8–11], practices in Asian countries, including Japan, have been generally undocumented. The purpose of this survey was to determine the current patterns of practice of and obstacles to implementation of 3D-IGBT use for cervical cancer in Japan.

## MATERIALS AND METHODS

In July 2012, a 30-item questionnaire (see Appendix) was sent to 171 Japanese facilities possessing high-dose-rate brachytherapy machines. Questions focused on the practice of brachytherapy for cervical cancer, plans for transitioning to 3D-IGBT, and the obstacles faced in implementation. Only one set of answers was allowed per facility to prevent redundancy. Non-responders received reminders in August 2012. A follow-up survey was sent in January 2015 to 38 facilities that had indicated plans to transition to 3D-IGBT in the first survey to query whether they had in fact followed through and, if so, which type of imaging modality was routinely used for treatment planning.

## RESULTS

### Respondents

Of 171 facilities, 144 (84%) responded to the questionnaire. Nine respondents did not use brachytherapy for gynecological malignancies; thus, 135 (78%) surveys were analyzed. Of these facilities, 34% treated 20–49 patients; 31% treated 10–19 patients; 19% treated <10 patients; 9% treated no patients; and 6% treated ≥50 patients, in 2011.

### Imaging modality

Ultrasound guidance for applicator insertion was used by 2% (3 of 135) of respondents routinely and 25% (34 of 135) occasionally when necessary; 73% (98 of 135) did not use it. With brachytherapy applicator insertion, X-ray films alone were acquired in 62% (84 of 135, Group 1) of the facilities. Various types of 3D images were acquired with applicator insertion in the remaining 51 facilities, 8 of which used 3D imaging exclusively. Table 1 lists the types of images acquired with applicator insertion as well as the imaging used for actual treatment planning. CT inside the brachytherapy room was available at 9 facilities. Of 43 facilities where X-ray films and CT or MRI were acquired with an inserted applicator, 29 facilities used X-ray films for actual treatment planning (Group 2) and 14 facilities used CT or MRI for treatment planning. Thus, 22 facilities in total used CT or MRI for treatment planning (Group 3). The most common answer regarding patient number in 2011 was 10–19 for Group 1, 20–49 for Group 2 and 20–49 for Group 3, respectively.

**Table 1.** Imaging used for image-guided brachytherapy (*n* = 135)

	Number	%
Imaging acquired with inserted applicator		
X-ray films alone	84	62
X-ray films + CT	41	30
X-ray films + CT + MRI	2	1
CT	7	5
CT + MRI	1	1
Imaging used for actual treatment planning		
X-ray films	113	84
CT	20	15
MRI	2	1

### 2D planning group not acquiring 3D imaging (Group 1)

On whether Group 1 (*n* = 84) was considering introducing a 3D treatment planning system within the next three years, 45% (38 of 84) of the facilities replied affirmatively. Among the positive respondents, 89% (34 of 38) intended to use CT for treatment planning, while the other 11% (4 of 38) intended to use both CT and MRI. In the follow-up survey, to which 31 of the original 38 facilities that had stated intentions to introduce a 3D planning system responded, 68% (21 of 31) indicated that they had begun acquiring CT images with applicator insertion, and 55% (17 of 31) used CT for treatment planning. Table 2 lists the reasons why 46 respondents indicated they were not considering a 3D treatment planning system.

**Table 2.** Reasons for using X-ray films in lieu of 3D imaging for treatment planning

	Number	%
Group 1 (*n* = 46)^a^		
geographically limited access to CT/MRI	17	37
applicators used were not CT/MRI-compatible	17	37
limited time for treatment planning	15	33
lack of knowledge for the planning	11	24
Group 2 (*n* = 29)^b^		
limited time for planning with CT/MRI	21	72
inadequate manpower	14	48
inadequate planning software	9	31
lack of knowledge for the planning	7	24
applicators used were not CT/MRI-compatible	6	21

^a^In Group 1, 46 of 84 facilities did not consider the introduction of a 3D-IGBT treatment planning system. ^b^In Group 2, all 29 facilities acquired CT but used X-ray films for treatment planning.

### 2D planning group acquiring 3D imaging (Group 2)

In Group 2 (*n* = 29), 31% of the facilities acquired 3D images via CT in every session, while 31% obtained images only for the first session. The remaining facilities used 3D imaging only when necessary. CT/MRI-compatible applicators and metal applicators were used in 17% and 83% of the facilities, respectively. The most common reason for acquiring 3D images in addition to X-ray films was visualizing the anatomical relationship between applicator and the organs at risk or the tumor (97%), followed by confirming perforation (52%), calculating DVH parameters after treatment (52%), and setting a secondary reference point alternative to Point A (24%). Table 2 lists the reasons for using X-ray films only for actual treatment planning. All 29 facilities chose CT and not MRI for acquiring 3D images. The reasons for using CT in lieu of MRI in Groups 1 and 2 are shown in Table 3.

**Table 3.** Reasons for using CT in lieu of MRI

	Number	%
Group 1 (*n* = 34)^a^		
geographically limited access to MRI	22	65
difficulty in ensuring reservations for MRI	14	41
applicators used were not MRI-compatible	13	38
inadequate manpower	11	32
long examination times	9	26
CT image is regarded as sufficient	9	26
Group 2 (*n* = 29)^b^		
difficulty in ensuring reservations for MRI	22	76
geographically limited access to MRI	20	69
applicators used were not MRI-compatible	12	41
inadequate manpower	11	38
long examination time	9	31
CT image was regarded as sufficient	7	24
Group 3 (*n* = 20)^c^		
difficulty in ensuring reservations for MRI	12	60
geographically limited access to MRI	8	40
applicators used were not MRI-compatible	6	30
CT image was regarded as sufficient	5	25
inadequate manpower	3	15
long examination time	3	15

^a^In Group 1, 34 of 84 facilities intended to use CT but not MRI for treatment planning in the future. ^b^In Group 2, all 29 facilities chose CT for acquiring 3D images. ^c^In Group 3, 20 of 22 facilities used CT for treatment planning.

### 3D planning group (Group 3)

In Group 3 (*n* = 22), 20 facilities used CT and not MRI for treatment planning, the reasons for which are shown in Table 3. Fifteen facilities (68%) acquired imaging by CT or MRI in every session, while 5 (23%) obtained images only for the first session. CT/MRI-compatible applicators and metal applicators were used in 6 (27%) and 16 (73%) of the facilities, respectively. For target dose specification, 21 facilities (95%) used Point A, while none of the facilities used HR-CTV alone. Table 4 lists the responses regarding detailed information on 3D treatment planning for cervical cancer.

**Table 4.** Summary of answers regarding 3D treatment planning for cervical cancer (*n* = 22)

Question	Answers (%)							
Which normal tissues do you routinely contour?	Not determined (18%)	Bladder (68%)	Rectum (73%)	Sigmoid colon (45%)	Small intestine (41%)	Vagina (5%)	Other (5%)	
Which targets do you routinely contour?	Not determined (27%)	GTV-BT (23%)	HR-CTV (45%)	IR-CTV (14%)	Other (5%)			
Which DVH parameter(s) do you routinely use?	HR-CTV D90 (36%)	HR-CTV D100 (36%)	HR-CTV D150 (0%)	HR-CTV D200 (5%)	V100 (9%)	V150 (0%)	V200 (0%)	Other (27%)
Which method do you use for dose specification to the target?	Point A (55%)	HR-CTV (0%)	Both (41%)	NR (5%)				
Is the treatment plan optimized whenever the CTV or GTV could not be fully covered by the prescribed dose?	Yes (77%)	No (14%)	NR (9%)					
If yes, what reference is used for the optimization?	Point A (23%)	HR-CTV D90 (45%)	Other DVH parameter (9%)	Other (14%)				
Which DVH parameters do you routinely use for the organs at risk?	D0.1cm^3^ (36%)	D1cm^3^ (32%)	D2cm^3^ (50%)	D5cm^3^ (9%)	Other (14%)			
Which method do you use for dose specification to the rectum?	ICRU points (27%)	DVH parameters (41%)	Both (27%)	NR (5%)				
Is optimization carried out using the rectal/sigmoid colon dose?	Yes (91%)	No (9%)						
If yes, what reference is used for the optimization?	ICRU points (23%)	D2cm^3^ (32%)	Other DVH parameter (5%)	Other (32%)	NR (9%)			
Which method do you use for dose specification to the bladder?	ICRU points (5%)	DVH parameters (36%)	Both (23%)	NR (36%)				
Is optimization carried out using the bladder dose?	Yes (55%)	No (45%)						
If yes, what reference was used for the optimization?	ICRU points (36%)	D2cm^3^ (32%)	Other DVH parameter (0%)	Other (9%)	NR (23%)			

GTV-BT = gross tumor volume at brachytherapy; CTV-BT = clinical target volume at brachytherapy; HR-CTV = high-risk clinical target volume; IR-CTV = intermediate-risk clinical target volume; D*n* = dose receiving *n* cm^3^ of HR-CTV from 90 to 200; V*n* = volume receiving *n*% of prescribed dose in the target; ICRU = International Commission on Radiation Units.

## DISCUSSION

According to our survey results, 3D imaging with brachytherapy applicator insertion was adopted in 38% (51 of 135) of the facilities in 2012, and was used for treatment planning in 16% (22 of 135). Two and a half years later, an additional 16% (21 of 135) and 13% (17 of 135) of the facilities had begun acquiring 3D images and adopting 3D image–based planning, respectively. Conversely, a separately published national survey in Japan reported that, of 816 linear accelerators in use in 2009, 81% and 41% were used for 3D conformal radiotherapy and intensity-modulated radiotherapy (IMRT), respectively, indicating a significant delay in the dissemination of 3D-IGBT [12]. Several study groups have reported the incidence of 3D planning using CT or MRI for gynecological brachytherapy, with a range of 50–74% (Table 5) [8–11]. Similarly, 3D planning for gynecological brachytherapy has lagged compared with external beam radiotherapy (EBRT) [13–14].

**Table 5.** Imaging modality for treatment planning in IGBT for cervical cancer

Study group	Year	Number	Responder	Response rate	X-ray films	CT	MRI
ABS [8]	2007	256	Physician	55%	43%	55%	2%
CANADA [9]	2009	58	Physician	62%	50%	45%	5%
UK [10]	2008→2011	45	Facility	96%	73%→26%	22%→53%	4%→21%
Australia & New Zealand [11]	2009	20	Facility	100%	30%	65%^a^	5%
The present study	2012	171	Facility	84%	84%	15%	1%

^a^One facility (5%) used CT in a 2D fashion to assess implant geometry. ABS = American Brachytherapy Society; IGBT = image-guided brachytherapy.

A recent study suggests that gynecological brachytherapy is underutilized in New South Wales, Western Europe and the USA [15]. In a Japanese study, 22% of cervical cancer patients were still not given brachytherapy during 2003–2005 [16]. Gill *et al.* also showed an increasing trend of EBRT boost utilization with IMRT and stereotactic body radiotherapy for cervical cancer [17]. Importantly, studies by Han *et al.* and Gill *et al.* demonstrated that combined use of EBRT and brachytherapy produced significantly better survival compared with EBRT alone, indicating that local brachytherapy offers a marked survival benefit in cervical cancer patients [17, 18]. Additionally, recent clinical reports on 3D-IGBT from single institutions have shown excellent local control (89–97%) as well as minimal late toxicities (2–8% of Grade 3 or worse) [7, 19, 20]. Thus, while EBRT boost ought not to be replaced, 3D-IGBT should be implemented alongside it in order to achieve maximum patient benefit.

In terms of obstacles to the implementation of 3D planning, both Group 1 and Group 2 reported similar deterrents (Table 3). MRI has been considered the gold standard for 3D-IGBT for cervical cancer because it provides more precise anatomical information; however, the transition to 3D planning with MRI has been slow (Table 5) [8–11]. Our survey showed that difficulty in ensuring reservations and geographically limited access were common reasons for using CT rather than MRI for treatment planning in Groups 1, 2 and 3 (Table 2).

At present, CT is most commonly used for IGBT treatment planning for cervical cancer (Table 5). Although a guideline for CT-based CTV in brachytherapy for cervical cancer was proposed independently of MRI-based HR-CTV, further evaluation of its feasibility and reliability will be necessary in the clinical setting [21]. Viswanathan *et al.* reported that CT-based tumor contours could significantly overestimate tumor width, resulting in significant differences in DVH parameters compared with MRI [22]. On the other hand, the accuracy of CT-based HR-CTV contouring was improved by adding information from 3D documentation of physical examinations [23]. In addition, a combination of MRI for the first fraction and subsequent CT-based planning is feasible when automatic applicator-based image registration and target transfer are technically available [24]. Therefore, the advantages and disadvantages of CT-based brachytherapy should be recognized, and efforts to minimize the uncertainty of contouring should be made at centers where the full use of MRI for treatment planning is limited.

Despite the small number of respondents, the present survey highlighted several insufficiencies concerning 3D-IGBT in Japan. First, the routine use of ultrasound for applicator insertion was only 2%, and occasional use was 24% in our survey. Second, we found that 23% of respondents obtained CT or MRI images only for the first session, while 68% acquired the images with each insertion. Finally, the relatively higher incidence of use of the D90 of HR-CTV was noted for plan optimization in a setting where CT was the main imaging modality. Originally, HR-CTV is a concept defined on MRI with no guidelines yet for contouring on CT. Using HR-CTV in facilities of limited MRI usage must be addressed.

In Group 3, Point A alone (55%) was most frequently used in our survey as a dose specification to the target, while 41% used both Point A and HR-CTV. In the ABS survey, Point A (76%) remained the most frequent prescription method [8]. The Canadian survey reported that 53% of respondents utilizing CT-based planning used Point A alone for dose prescription and 20% used both Point A and the HR-CTV [9]. The UK survey demonstrated that 93% of centers in both 2008 and 2011 provided their standard prescription for EBRT and brachytherapy dose to Point A [10]. Thus, even in the era of 3D-IGBT, together with the concept of CTV, Point A still has an important role for dose specification to the target. In conclusion, limited time and inadequate manpower were major barriers to the implementation of 3D-IGBT. Based on the promising clinical outcomes reported in the literature, removing these barriers and expanding the adoption of 3D-IGBT should be prioritized.

## FUNDING

Funding to pay the Open Access publication charges for this article was provided by a Grant-in-Aid for Clinical Cancer Research, Health and Labour Sciences Research Grants from the Japanese Ministry of Health, Labour, and Welfare [10103757] and for KAKENHI from the Japan Society for the Promotion of Science [26461879].

## Appendix

1. Please state which of the following hospitals are you associated with?

 1) Prefectural or Metropolitan Designated Cancer Center or Hospital; 2) Regional Designated Cancer Center or Hospital; 3) Other ( )

2. How many new cases of intracavitary irradiation for cervical cancer did your institution have in the year 2011?

 1) N/A; 2) Less than 10; 3) 10–19; 4) 20–49; 5) 50 or more

**This question relates to analysis of the circumstances surrounding image-guided three-dimensional treatment planning for cervical cancer.**

**The question deals with both applicator insertion and image acquisition.**

3. Is ultrasonography used for applicator insertion?

 1) Always; 2) If necessary; 3) No

4. What imaging modality is acquired with insertion of the applicator? (Please mark all applicable answers)

 1) X-ray films; 2) CT in the treatment room; 3) CT outside the treatment room; 4) MRI; 5) Ultrasound

5. What imaging modality is used for the actual treatment planning?

 1) X-ray films; 2) CT; 3) MRI; 4) Ultrasound

**The following questions are categorized into three groups.**

Group 1: If only response 1) was selected for questions 4 and 5

 ⇒ The survey ends after questions 6 through 9 below are answered.

Group 2: If response numbers 2), 3), 4), or 5) are selected for question 4 above and response 1) is selected for question 5 above

 ⇒ The survey ends after questions 10 through 14 below are answered.

Group 3: If responses 2), 3), 4), or 5) are selected for question 4 and responses 2), 3), or 4) are selected for question 5

 ⇒ The survey ends after questions 15 through 31 are answered.

**Questions for Group 1**

The 2012 Health and Labor Sciences Research Grant Cancer Clinical Research Projects “Research relating to promotion and quality control of radiotherapy contributing to eliminating cancer care disparities” Team

6. Are you considering introducing a three-dimensional treatment planning system within the next three years?

 1) Yes; 2) No

7. This question is to be answered only by those hospitals that selected response 1) in question 6: What imaging modality do you intend to use for treatment planning? (Please mark all applicable answers)

 1) CT; 2) MRI; 3) Other ( )

8. This question is to be answered only by those hospitals that selected response 1) in question 7: What is the reason for using CT and not MRI for treatment planning? (Please mark all applicable answers)

  1) Difficulty in ensuring reservations for MRI; 2) Geographically limited access to MRI; 3) Long examination times; 4) Inadequate manpower; 5) Applicators used were not MRI-compatible; 6) CT examination is regarded as sufficient; 7) Other ( )

9. This question is to be answered only by those hospitals that selected response 2) in question 6: Please state the reason for not considering the introduction of a three-dimensional treatment planning system? (Please mark all applicable answers)

  1) Did not consider it to be necessary; 2) Lack of evidence; 3) Limited time for treatment planning; 4) Inadequate manpower; 5) Lack of knowledge for the planning (target/OAR definitions, calculations, etc.); 6) Applicators used were not CT/MRI-compatible; 7) Insufficient planning software; 8) Geographically limited access to the CT/MRI; 9) Not covered by national insurance system; 10) Other ( )

  This concludes the questionnaire for Group 1. Thank you for your participation.

**Questions for Group 2**

10. How often is CT or MRI imaging performed with insertion of the applicator?

 1) Every session; 2) Only the first session; 3) Other ( )

11. What applicator do you use?

 1) CT/MRI-compatible applicator; 2) Metal applicator

12. What is the reason for acquiring images other than X-ray films? (Please mark all applicable answers)

 1) To confirm the perforation; 2) To see the anatomical relationship between the applicator and the organ at risk or tumor; 3) To set a reference point other than Point A; 4) To calculate DVH parameters after treatment; 5) Other ( )

13. State the reason for using X-ray films for the actual treatment planning? (Please mark all applicable answers)

 1) Lack of evidence for planning other than the X-ray films; 2) Limited time for planning with CT/MRI; 3) Inadequate manpower; 4) Lack of knowledge for the planning (target/OAR delineation, calculations, etc.); 5) Applicators used were not CT/MRI-compatible; 6) Inadequate planning software; 7) Other ( )

14. This question is to be answered only by those hospitals that selected CT in question 4: What is the reason for using CT and not MRI? (Please mark all applicable answers)

 1) Difficulty in ensuring reservations for MRI; 2) Geographically limited access to MRI; 3) Long examination time; 4) Inadequate manpower; 5) Applicators used were not MRI-compatible; 6) CT is regarded as sufficient; 7) Other ( )

  This concludes the questionnaire for Group 2. Thank you for your participation.

**Questions for Group 3**

15. This question is to be answered only by those hospitals that selected CT in question 4: What is the reason for using CT and not MRI in treatment planning? (Please tick all applicable answers)

 1) Difficulty in ensuring reservations for MRI; 2) Geographically limited access to MRI; 3) Long examination time; 4) Inadequate manpower; 5) Applicators used were not MRI-compatible; 6) CT is regarded as sufficient; 7) Other ( )

16. How often is CT or MRI imaging performed with insertion of the applicator?

 1) Every session; 2) Only the first time; 3) Other ( )

The 2012 Health and Labor Sciences Research Grant Cancer Clinical Research Projects “Research relating to promotion and quality control of radiotherapy contributing to eliminating cancer care disparities” Team

17. Which applicator do you use?

 1) CT/MRI-compatible applicator; 2) Metal applicator

18. Which normal tissues do you routinely contour? (Please tick all applicable answers)

 1) Not determined; 2) Bladder; 3) Rectum; 4) Sigmoid colon; 5) Small intestine; 6) Vagina; 7) Other ( )

19. Which targets do you routinely contour? (Please tick all applicable answers)

 1) Not determined; 2) GTV-BT; 3) HR-CTV; 4) IR-CTV; 5) Other ( )

20. Which DVH parameter(s) do you routinely use? (Please tick all applicable answers)

 1) D90; 2) D100; 3) D150; 4) D200; 5) V100; 6) V150; 7) V200; 8) Other

21. Which method do you use for dose specification to the target?

 1) Point A; 2) HR-CTV; 3) Both

22. Is the treatment plan optimized whenever the CTV or GTV could not be fully covered by the prescribed dose?

 1) Yes; 2) No

23. If yes, what reference is used for the optimization?

 1) Point A; 2) HR-CTV D90; 3) Other DVH parameter; 4) Other

24. Which DVH parameters do you routinely use for the organs at risk? (Please tick all applicable answers)

 1) D0.1cc; 2) D1cc; 3) D2cc; 4) D5cc; 5) Other (––––––––––––––––––––––––)

25. Which method do you use for dose specification to the rectum?

 1) ICRU points; 2) DVH parameters; 3) Both

26. Is optimization carried out using the rectum/sigmoid colon dose?

 1) Yes; 2) No

27. If yes, what reference is used for the optimization?

 1) ICRU points; 2) D2cc; 3) Other DVH parameter; 4) Other

28. Which method do you use for dose specification to the bladder?

 1) ICRU points; 2) DVH parameters; 3) Both

29. Is optimization carried out using the bladder dose?

 1) Yes; 2) No

30. If yes, what reference was used for the optimization?

 1) ICRU points; 2) D2cc; 3) Other DVH parameter; 4) Other

  This concludes the questionnaire for Group 3. Thank you for your participation.
